# Reduced Gonadotrophin Receptor Expression Is Associated with a More Aggressive Ovarian Cancer Phenotype

**DOI:** 10.3390/ijms22010071

**Published:** 2020-12-23

**Authors:** Janelle Cheung, Noor A. Lokman, Riya D. Abraham, Anne M. Macpherson, Eunice Lee, Frank Grutzner, Nicolae Ghinea, Martin K. Oehler, Carmela Ricciardelli

**Affiliations:** 1 Adelaide Medical School, Robinson Research Institute, University of Adelaide, Adelaide, SA 5000, Australia; janellecheung24@gmail.com (J.C.); noor.lokman@adelaide.edu.au (N.A.L.); abrahamriya00@gmail.com (R.D.A.); anne.macpherson@adelaide.edu.au (A.M.M.); martin.oehler@adelaide.edu.au (M.K.O.); 2 School of Biological Science, Robinson Research Institute, University of Adelaide, Adelaide, SA 5005, Australia; eunicehsiuyee.lee@adelaide.edu.au (E.L.); frank.grutzner@adelaide.edu.au (F.G.); 3 Curie Institute, Research Center, Translational Research Department, Tumor Angiogenesis Team, 75005 Paris, France; nicolae.ghinea@curie.fr; 4 Royal Adelaide Hospital, Adelaide, SA 5000, Australia

**Keywords:** ovarian cancer, serous ovarian cancer, gonadotropins, FSHR, LHCGR, cancer progression

## Abstract

Follicle-stimulating hormone (FSH) and luteinising hormone (LH) play important roles in regulating cell growth and proliferation in the ovary. However, few studies have explored the expression of FSH and LH receptors (FSHR and LHCGR) in ovarian cancer, and their functional roles in cancer progression remain inconclusive. This study investigated the potential impact of both mRNA (*FSHR*, *LHCGR*) and protein (FSHR, LHCGR) expression on ovarian cancer progression using publicly available online databases, qRT-PCR (high grade serous ovarian cancers, HGSOC, *n* = 29 and benign ovarian tumors, *n* = 17) and immunohistochemistry (HGSOC, *n* = 144). In addition, we investigated the effect of *FSHR* and *LHCGR* siRNA knockdown on the pro-metastatic behavior of serous ovarian cancer cells in vitro. High *FSHR* or high *LHCGR* expression in patients with all subtypes of high-grade ovarian cancer was significantly associated with longer progression-free survival (PFS) and overall survival (OS). High FSHR protein expression was associated with increased PFS (*p* = 0.050) and OS (*p* = 0.025). HGSOC patients with both high FSHR and high LHCGR protein levels had the best survival outcome, whilst both low FSHR and low LHCGR expression was associated with poorest survival (*p* = 0.019). Knockdown of FSHR significantly increased the invasion of serous ovarian cancer cells (OVCAR3 and COV362) in vitro. LHCGR knockdown also promoted invasion of COV362 cells. This study highlights that lower FSHR and LHCGR expression is associated with a more aggressive epithelial ovarian cancer phenotype and promotes pro-metastatic behaviour.

## 1. Introduction

Ovarian cancer is the leading cause of cancer-related mortality among gynaecological cancers in the Western world [1]. Approximately 21,750 new cases and 13,940 deaths due to ovarian cancer are expected in 2020 in the United States [1]. Epithelial ovarian cancer (EOC) accounts for 90% of diagnosed cases and up to 68% of those are of a serous epithelial subtype [2]. Other subtypes include mucinous, clear cell, and endometrioid ovarian cancers [2]. The lack of specific symptoms and effective methods for early detection are the reason that 75% of patients are diagnosed with advanced metastatic intraabdominal disease (Federation International of Gynaecology and Obstetrics, FIGO stage III and IV) and have very poor prognosis [2]. Despite aggressive treatments including radical debulking surgery and chemotherapy, the majority of patients relapse and ultimately die from chemotherapy resistant disease [3]. The glycoprotein hormones follicle-stimulating hormone (FSH) and luteinising hormone (LH) are gonadotropins produced by gonadotrope cells in the anterior pituitary gland [4]. FSH and LH regulate the secretion of sex steroid hormones from the gonads by binding to their specific receptors, follicle-stimulating hormone receptor (FSHR) and luteinising hormone receptor (LHCGR), respectively [5,6]. In females, FSHR is predominantly expressed on the granulosa and ovarian surface epithelium (OSE) and when activated induces follicular growth and development, as well as the secretion of estrogen and the conversion of androgen to estrogen [5,6,7]. LHCGR is also expressed by the granulosa and OSE, in addition to corpora lutea and theca cells, and stimulates ovulation, development of the corpus luteum and estrogen progesterone and androgen production [6,7]. The balance of gonadotropin levels in females is maintained by the negative feedback loop regulated by sex steroid hormones and inhibin [8]. However, in postmenopausal women, sex steroid hormone production is reduced and the lack of negative feedback on FSH and LH production can lead to a chronic elevation of these hormones [9]. Post-menopausal women have up to 18.4-fold higher FSH levels and 3.4-fold higher LH levels compared to pre-menopausal women [10]. The ‘gonadotropin theory’ proposes that chronically elevated FSH and LH levels may lead to increased activation of FSHR and LHCGR, respectively, and subsequently stimulate ovarian cancer proliferation [11]. As 75% of cases occur in post-menopausal women, elevated gonadotropin levels may be associated with ovarian cancer development [9].

FSHR and LHCGR belong to a subgroup of G-protein coupled receptors with seven transmembrane helices. These receptors are characterized by a large ectodomain containing several leucine-rich repeats where FSH and LH bind [12,13]. These receptors are involved in mediating cell growth in the ovary, and the activation of their signaling pathways have been linked to neoplasia [7]. *FSHR* is expressed in up to 50% of high grade serous ovarian cancer (HGSOC) [14], however, limited studies have investigated the functional role of FSHR expression in ovarian cancer progression. Increased *FSHR* mRNA [15] and protein expression have been linked with low tumor grade in serous carcinomas [16] and reduced overall survival (OS) [17]. Decreased LHCGR protein expression [16] and *LHCGR* mRNA expression were associated with high tumor grade [18] and reduced patient survival in EOC [17,19].

To date few studies have explored the effects of FSHR knockdown and none have investigated *LHCGR* knockdown on human ovarian cancer cell behavior. This study investigated whether *FSHR* and *LHCGR* mRNA and protein levels are associated with ovarian cancer progression and if the knockdown of these gonadotrophin receptors affects invasive behavior of serous ovarian cancer cells in vitro.

## 2. Results

### 2.1. FSHR and LHCGR Expression Are Associated with Tumor Stage and Tumor Grade

**Figure 1:**
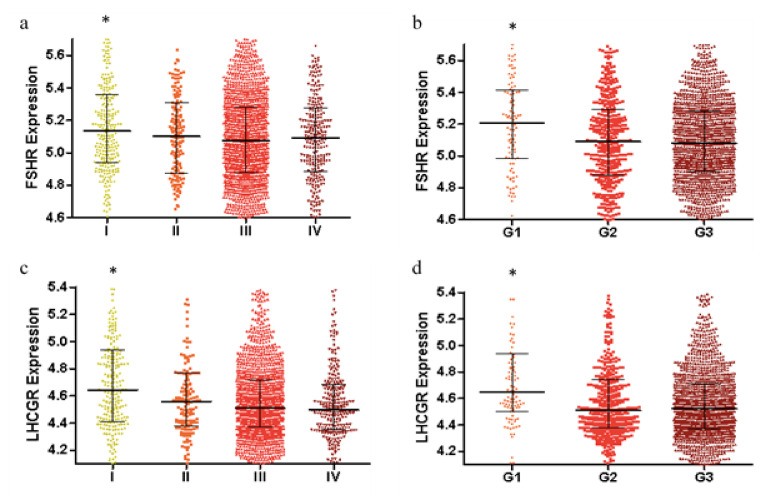
*FSHR* and *LHCGR* mRNA expression is increased in early stage and low-grade ovarian cancers: (**a**) *FSHR* expression in stage I, II, III and IV ovarian cancers; (**b**) *FSHR* expression in grade I, II and III ovarian tumors; (**c**) *LHCGR* expression in stage I, II, III and IV ovarian cancers. (**d**) *LHCGR* expression in grade I, II and III ovarian cancers. Total *n* = 3431 from CSIOVDB microarray gene expression database [20]. * *p* < 0.05 compared to the rest, Mann–Whitney U test from http://csibio.nus.edu.sg/CSIOVDB/CSIOVDB.html.

**Figure 2:**
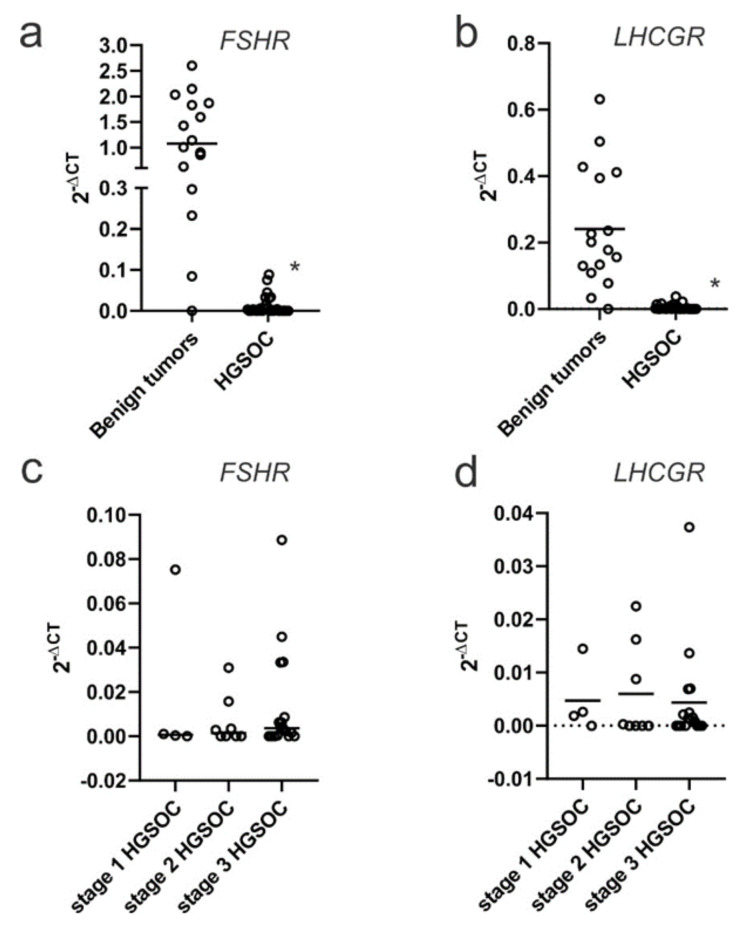
*FSHR* and *LHCGR* mRNA expression is reduced in high grade serous ovarian carcinoma (HGSOC) compared to benign serous ovarian tumors: (**a**) *FSHR* in benign ovarian tumor (*n* = 17) and HGSOC (*n* = 29); (**b**) *LHCGR* expression in benign ovarian tumors (*n* = 17) and HGSOC (*n* = 29). (**c**) *FSHR* expression in FIGO stage I (*n* = 4), FIGO II (*n* = 8) and FIGO III (*n* = 17) HGSOC. (**d**) *LHCGR* expression in stage I (*n* = 4), II (*n* = 8) and III (*n* = 17) HGSOC. * *p* < 0.05, Mann–Whitney U test. Circles are data from each patient.

Using the publicly available CSIOVDB database, *FSHR* and *LHCGR* expression was increased in early-stage ovarian cancer (stage I) compared to stage II, III or IV cancers (*p* < 0.01, Figure 1a,c). Similarly, *FSHR* and *LHCGR* expression was increased in low-grade ovarian cancers (grade I), compared to high grade ovarian cancers (grade II or grade III, *p* < 0.01, Figure 1b,d). We additionally observed a significant reduction in the expression of both *FSHR* (Figure 2a) and *LHCGR* (Figure 2b) in HGSOC compared to benign serous ovarian tumors. We did not find any relationship between tumor stage and *FSHR* (Figure 2c) or *LHCGR* expression in the HGSOC tumor cohort (Figure 2d).

### 2.2. Reduced FSHR and LHCGR mRNA Expression Is Associated with Poor Patient Outcome

**Figure 3:**
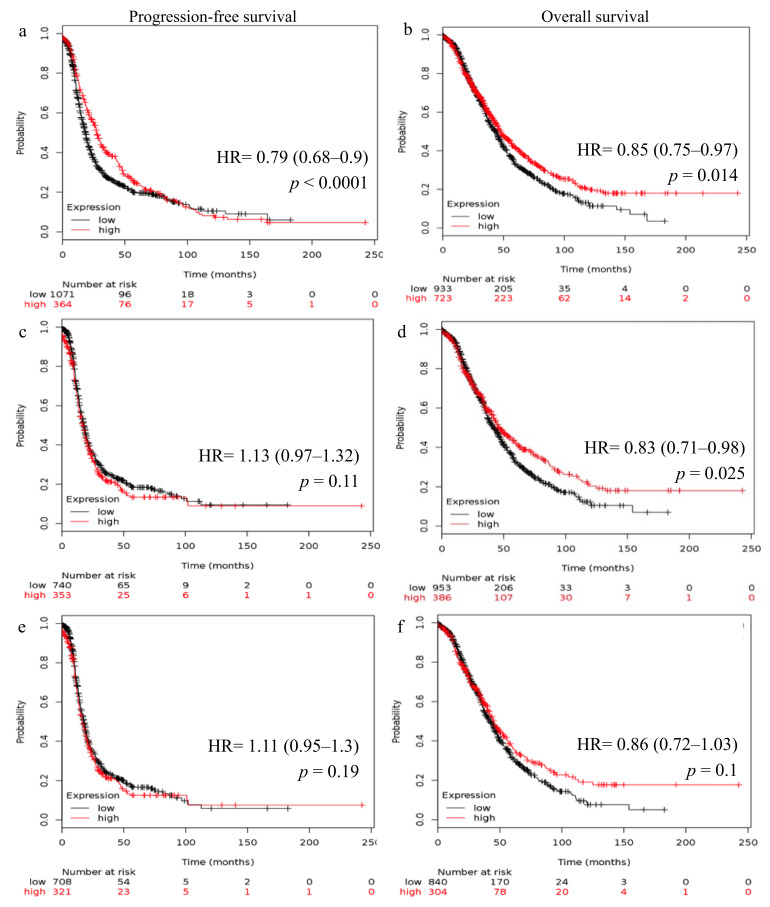
Kaplan–Meier survival analysis showing association of *FSHR* mRNA expression with ovarian cancer patient outcome. (**a***) FSHR,* all ovarian cancers (*n* = 1435)*,* progression-free survival (PFS)*. (***b**) *FSHR*, all ovarian cancers (*n* = 1656), overall survival (OS). (**c**) *FSHR*, high-grade ovarian cancers, (*n* = 1093), PFS. (**d**) *FSHR*, high-grade ovarian cancers, (*n* = 1339), OS. (**e**) *FSHR*, high-grade serous ovarian cancers, (*n* = 1029), PFS. (**f**) *FSHR*, high-grade serous ovarian cancers (*n* = 1144), OS. Patients were grouped using the best cut-off and hazard ratio (HR), 95% confidence intervals, log-rank test, *p* values were calculated by the Kaplan-Meier online plotter tool [21].

**Figure 4:**
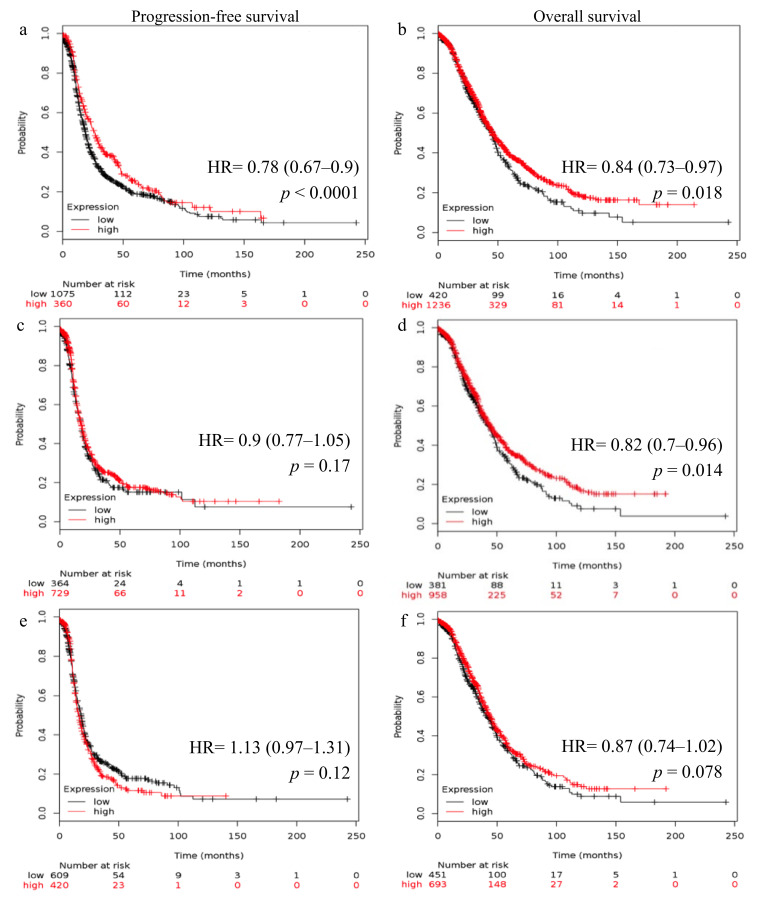
Kaplan Meier survival analysis showing association of *LHCGR* mRNA expression with ovarian cancer patient outcome. (**a**) *LHCGR*, all ovarian cancers (*n* = 1435), progression-free survival (PFS). (**b**) *LHCGR*, all ovarian cancers (*n* = 1656), overall survival (OS). (**c**) *LHCGR*, high-grade ovarian cancers, (*n* = 1093), PFS. (**d**) *LHCGR*, high-grade ovarian cancers, (*n* = 1339), OS. (**e**) *LHCGR*, serous high-grade ovarian cancers, (*n* = 1029), PFS. (**f**) *LHCGR*, serous high-grade ovarian cancers (*n* = 1144), OS. Patients were grouped using the best cut-off and hazard ratio (HR), 95% confidence intervals and log-rank *p* were calculated by the Kaplan–Meier online plotter tool [21].

Survival curves generated using the Kaplan–Meier online plotter showed the relationship between *FSHR* and *LHCGR* expression and patient outcome. High *FSHR* expression in all ovarian cancer patients was linked with higher progression-free survival (PFS, Hazard ratio, HR, 0.79; 95% CI 0.68–0.9, *p* < 0.0001, Figure 3a) and OS (HR 0.85; 95% CI 0.75–0.97, *p* = 0.014, Figure 3b). High *FSHR* expression was also associated with higher OS in patients with high-grade ovarian cancer (HR 0.83; 95% CI 0.71–0.98, *p* = 0.025, Figure 3d). Similarly, high *LHCGR* expression was associated with higher PFS (HR 0.78; 95% CI 0.67–0.9, *p* < 0.0001, Figure 4a) and OS (HR 0.84; 95% CI 0.73–0.97, *p* = 0.018, Figure 4b) in all ovarian cancers and was linked with higher OS in patients with high-grade ovarian cancer (HR 0.82; 95% CI 0.7–0.96, *p* = 0.014, Figure 4d). No significant difference in *FSHR* expression (Figure 3c) and *LHCGR* expression (Figure 4c) was found with PFS in patients with high-grade ovarian cancer. No significant relationship was also observed between either *FSHR* (Figure 3e,f) or *LHCGR* (Figure 4e,f) expression and PFS or OS in patients with HGSOC.

### 2.3. FSHR Protein Expression Is Associated with Patient Outcome

**Figure 5:**
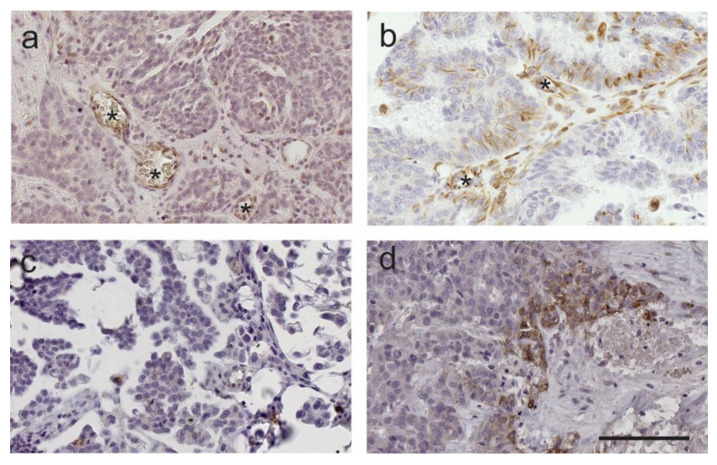
Examples of FSHR and LHCGR immunostaining in serous ovarian cancer cells. follicle-stimulating hormone receptor (FSHR) 323 (1/300 obtained from Prof. Nicolae Ghinea and LHCGR H-50 (1/200, Santa Cruz Biotechnology, Dallas, TX, USA) (**a**) low FSHR 323 immunostaining in cancer cells, (**b**) high FSHR 323 immunostaining in cancer cells, (**c**) low LHCGR immunostaining in cancer cells, (**d**) high LHCGR immunostaining in cancer cells. Scale bar = 100 µm (all images same magnification). * indicates FSHR positive blood vessels.

FSHR expression was observed in cancer cells, tumor associated stroma and blood vessels (Figure 5a,b), whilst focal LHCGR expression was present predominately in the tumor cells (Figure 5c,d). Ovary and fallopian tube from pre-menopausal women used as positive controls showed FSHR and LHCGR expression in granulosa cells and secretory cells in the fallopian tube epithelium (Figure S2). No staining was observed when mouse IgG or rabbit IgG was substituted for the primary antibodies (Figure S2). Examples of low and high FSHR and LHCGR immunostaining in HGSOC tissues are shown in Figure 5. High FSHR IR (≥3) and high LHCGR IR (≥3) expression were observed in 65.2% (73/112) and 31.7% (38/120) of HGSOC tissues examined, respectively. FSHR IR score was not associated with clinical and pathological parameters in the TMAs cohort including patient’s age (*p* = 1.00), FIGO stage (*p* = 0.20), tumor grade (*p* = 0.59) or the presence of residual disease (*p* = 1.000) (Table S5). LHCGR IR score was not associated with patient’s age (*p* = 0.84), FIGO stage (*p* = 0.73), tumor grade (*p* = 0.18) but significantly associated with the presence of residual disease (*p* = 0.01) (Table S5).

**Figure 6:**
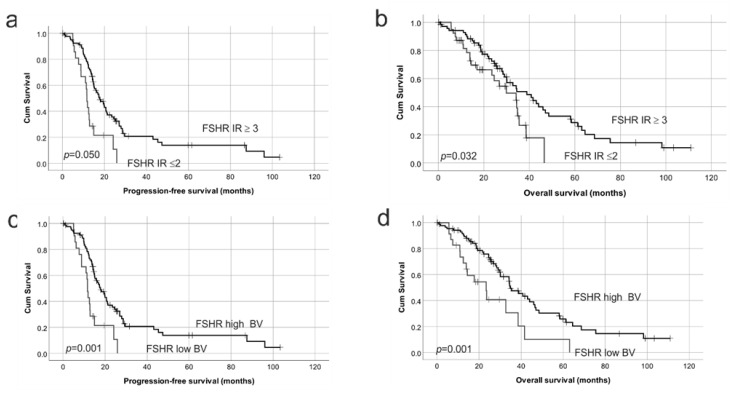
Kaplan–Meier survival analysis showing the relationship between FSHR protein expression with progression-free survival (PFS) and overall survival (OS) in HGSOC. (**a**) FSHR immunoreactive score (IR) in cancer cells with PFS; FSHR IR ≥ 3 (relapse; 52/69 patients), FSHR IR ≤ 2 (relapse; 27/33 patients) (**b**) FSHR IR score in cancer cells with OS, FSHR IR ≥ 3 (ovarian cancer death; 46/71 patients), FSHR IR ≤ 2 (ovarian cancer death; 22/39 patients). (**c**) FSHR IR score in blood vessels (BVs) with PFS, FSHR high BV (relapse; 61/81 patients), FSHR low BV (relapse; 18/21 patients). (**d**) FSHR IR score in BVs with OS, FSHR high BV (ovarian cancer death; 51/87 patients), FSHR low BV (ovarian cancer death; 17/23 patients). Log rank test.

**Figure 7:**
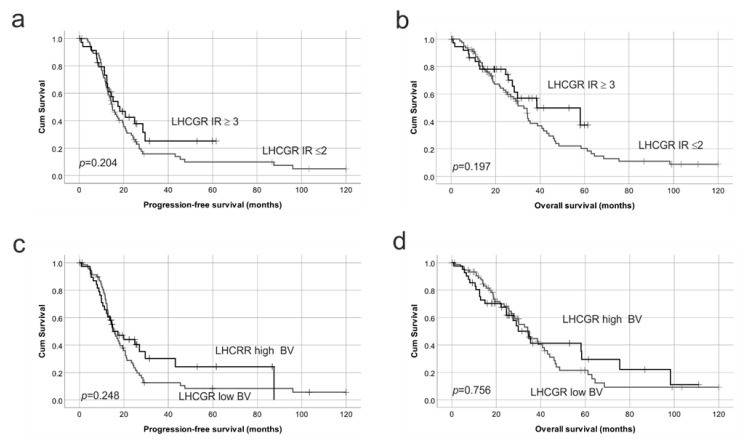
Kaplan–Meier survival analysis showing the relationship between LHCGR protein expression with progression-free survival and overall survival in HGSOC. (**a**) LHCGR immunoreactive score (IR) in cancer cells with PFS; LHCGR IR ≥ 3 (relapse; 63/74 patients), LR IR ≤ 2 (relapse; 21/35 patients) (**b**) LHCGR IR score in cancer cells with OS, LHCGR IR ≥ 3 (ovarian cancer death; 15/38 patients), LR IR ≤ 2 (ovarian cancer death; 57/81 patients). (**c**) LHCGR IR score in blood vessels (BVs) with PFS, LHCGR high BV (relapse; 66/39 patients), LHCGR low BV (relapse; 57/69 patients). (**d**) LHCGR IR score in BVs with OS, LHCGR high BV (ovarian cancer death; 24/42 patients), LHCGR low BV (ovarian cancer death; 47/75 patients). Log rank test.

Patients with low FSHR IR (≤2) had significantly reduced PFS (*p* = 0.05, Figure 6a) and OS (*p* = 0.03, Figure 6b) compared to patients with high FSHR IR (≥3). Interestingly, low FSHR blood vessel positivity (≤1) was also associated with reduced PFS (*p* = 0.001, Figure 6c) and OS (*p* = 0.001, Figure 6d). Univariate Cox Regression analysis showed no significant relationship with age, FIGO stage or tumor grade and PFS or OS (Table 1a). However, the presence of residual disease was associated with a 2.4-fold increased risk of ovarian cancer death (*p* = 0.024). Cox regression analysis confirmed that high FSHR IR score in cancer cells (Relative risk, RR = 0.55, *p* = 0.035) and FSHR positive blood vessels (RR = 0.40, *p* = 0.001) were significantly associated with a reduced risk of ovarian cancer death (Table 1a). No significant relationship was observed between PFS or OS and LHCGR IR in tumor cells or LHCGR positivity in blood vessels (BVs, Figure 7, Table 1).

### 2.4. Reduced Protein Expression of Both FSHR and LHCGR Are Associated with Poor Patient Outcome

**Figure 8:**
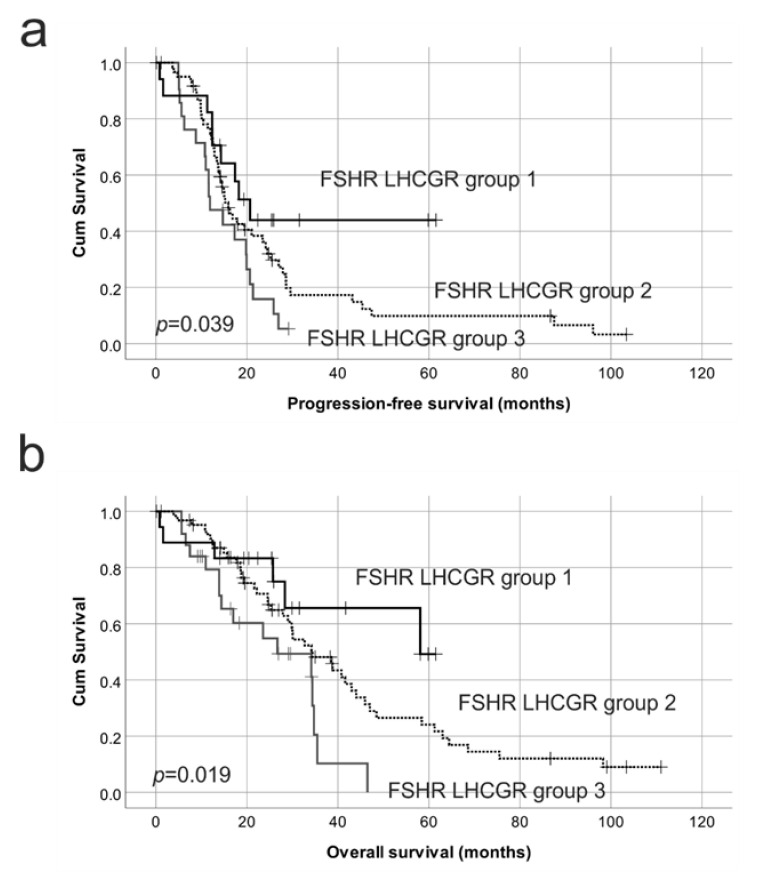
Kaplan–Meier survival analysis showing the relationship with combined FSHR and LHCGR protein expression with progression-free survival (PFS) and overall survival (OS). (**a**) Combined FSHR and LHCGR immunoreactive score groups with PFS. Group 1 = FSHR IR ≥3 and LHCGR IR ≥3 (relapse; 9/18 patients); Group 2 = FSHR IR ≥3 and LHCGR ≤2 or FSHR IR ≤2 and LHCGR IR ≥3 (relapse; 49/61 patients); Group 3 = FSHR IR ≤2 and LHCGR IR ≤2 (relapse; 19/21 patients) (**b**) Combined FSHR and LHCGR immunoreactive score groups with OS. Group 1 = FSHR IR ≥3 and LHCGR IR ≥3 (ovarian cancer death; 6/19); Group 2 = FSHR IR ≥3 and LHCGR ≤2 or FSHR IR ≤2 and LHCGR IR ≥3 (ovarian cancer death; 44/64); Group 3 = FSHR IR ≤2 and LHCGR IR ≤2 (ovarian cancer death; 16/25).

We investigated the relationship of both FSHR and LHCGR protein expression with patient outcome by combining patient groups with different levels of FSHR and LHCGR protein expression. These are: group 1 with both high FSHR and high LHCGR (FSHR IR ≥3 and LHCGR IR ≥3), group 2 with either high FSHR IR and low LHCGR IR or low FSHR and high LHCGR IR (FSHR IR ≥3 and LHCGR ≤2 or FSHR IR ≤2 and LHCGR IR ≥3) and group 3 with both low FSHR and low LHCGR (FSHR IR ≤2 and LHCGR IR ≤2). Group 3 patients had the worst outcome for both PFS and OS whilst group 1 patients had significantly higher PFS and OS (Table 1a, Figure 8). Group 2 patients had an intermediate risk of progression or death (Table 1, Figure 8). In a multivariable Cox regression analysis for PFS, which included FSHR BV positivity and the combined FSHR and LHCGR groups, only FSHR BV positivity remained an independent predictor for PFS (RR = 0.37, *p* = 0.002. Table 1b). In the multivariable Cox regression analysis for OS with residual disease, FSHR BV positivity and combined FSHR and LHCGR patient groups, the presence of residual disease (RR 3.06, *p* = 0.021), FSHR positive BVs (RR = 0.48, *p* = 0.020) and both a high FSHR and high LHCGR IR score (Group 3) remained independent predictors of OS (RR = 0.50, *p* = 0.044, Table 1b).

### 2.5. Characterization of Gonadotropin Receptor Expression in Serous Ovarian Cancer Cell Lines

**Figure 9:**
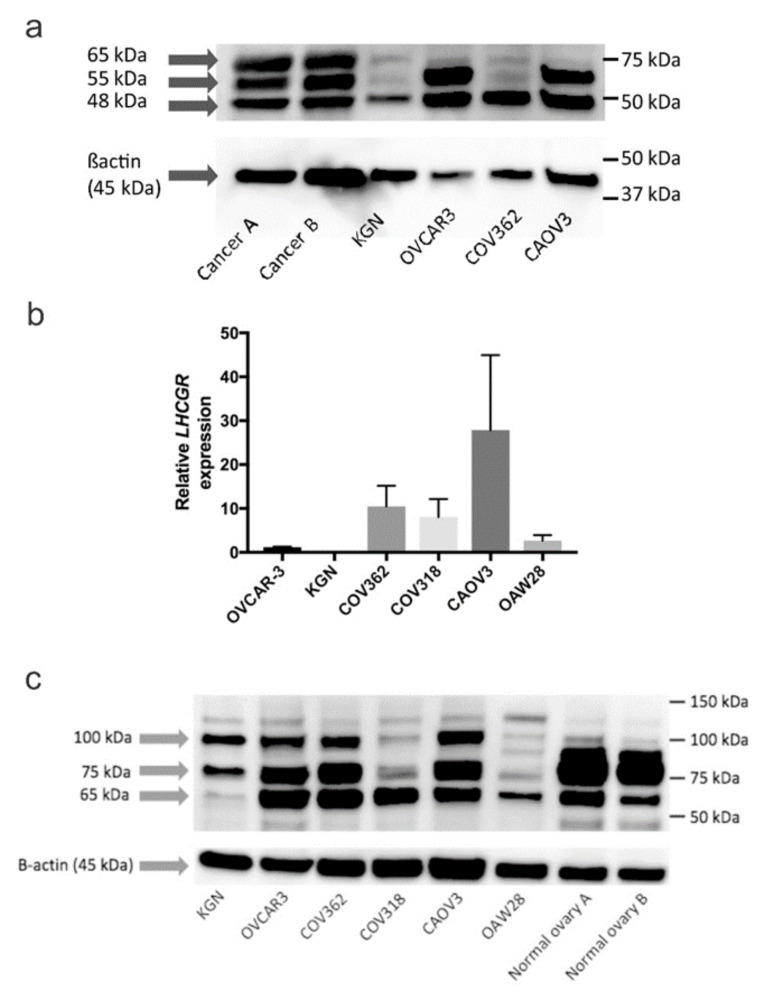
Follicle-stimulating hormone receptor (FSHR) and luteinising hormone receptor (LHCGR) expression in serous ovarian cancer cell lines. (**a**) Protein extracts from cell lines (~40 µg) and high-grade ovarian cancer tissues (~5 µg) were electrophoresed and immunoblotted with FSHR H-190 (1/400, Santa Cruz) antibody and β-actin (1/2000, Abcam, Cambridge, MA, USA) was used as a loading control. Bands were detected at ~48 kDa, ~55 kDa and ~65 kDa. (**b**) *LHCGR* mRNA expression in ovarian cancer cell lines using quantitative real-time PCR. Relative expression was normalised to the house keeping gene β-actin using the 2^−∆∆CT^ method. Data is expressed as the mean ± SEM from three individual RNA samples obtained from 2 independent experiments. (**c**) Protein extracts from cell lines (~40 µg) and normal ovaries (~5 µg) were electrophoresed and immunoblotted with LHCGR antibody (LS-C3345991/1000, LifeSpan Biosciences, Seattle, WA, USA) antibody and β-actin (1/2000, Abcam) was used as a loading control. Major bands were detected at ~65 kDa, ~75 kDa and ~100 kDa.

FSHR and LHCGR mRNA and protein expression were assessed in a range of human ovarian cancer cell lines by qRT-PCR and western blotting, respectively. *FSHR* mRNA expression was found in KGN granulosa tumor cell line and positive control human ovary tissue but was undetectable in the serous ovarian cancer cell lines. An FSHR antibody (H-190, Santa Cruz Biotechnology) detected protein bands at ∼48 kDa, ∼55 kDa and ∼65 kDa in the serous ovarian cancer tissue extracts shown to express FSHR in immunohistochemistry (Figure 9a). A strong band at ∼48 kDa was detected in protein extracts from all cell lines including the positive control cell line, KGN (Figure 9a). Weaker bands at ∼55 kDa and ∼65 kDa were detected in protein extracts from KGN and COV362 cells (Figure 9a). Strong bands at ∼55 kDa were also detected in protein extracts from OVCAR3 and CAOV3 cells (Figure 9a). Similar bands at ∼65 kDa and ∼50 kDa were also observed using the FSHR antibody (FSHR 323, 1/600) used in the immunohistochemistry study (Figure S3). We observed no bands in the protein extract prepared from mouse cumulus oocyte complexes (COCs) which was the negative control for the FSHR 323 antibody (Figure S3).

*LHCGR* mRNA expression was observed in all cell lines except KGN cells (Figure 9b). The highest *LHCGR* expression was observed in CAOV3 cells (Figure 9b). An LHCGR antibody (LS-C334599, Sapphire Bioscience) detected major bands at ∼65 kDa, ~75 kDa and ∼100 kDa in all serous ovarian cancer cell lines (Figure 9c). Protein bands at ∼75 kDa and ∼100 kDa were also observed in KGN cells (Figure 9c). Major protein bands at ∼65 kDa, ∼75 kDa and ∼80 kDa (doublet) were observed in tissue extracts from normal human ovary (Figure 9c). Two serous ovarian cancer cell lines, OVCAR3 and COV362, which expressed both FSHR and LHCGR, were selected for the gonadotropin receptor knockdown and invasion studies below.

### 2.6. The Effects of FSHR and LHCGR Knockdown on Serous Ovarian Cancer Invasion In Vitro

**Figure 10:**
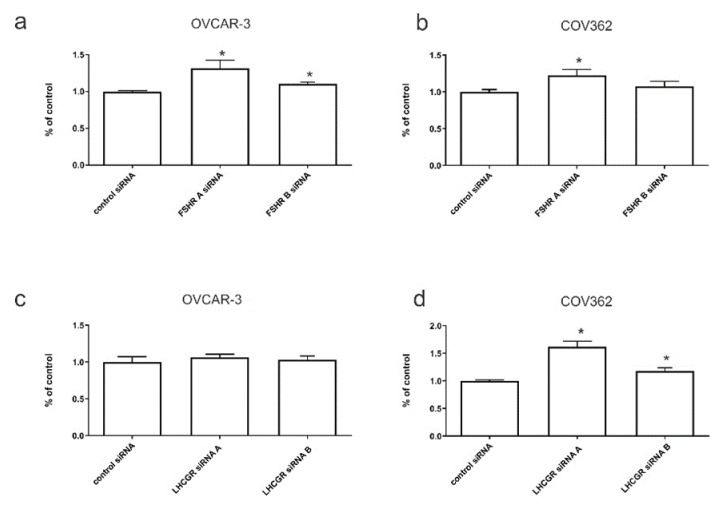
Effects of FSHR and LHCGR knockdown on serous ovarian cancer cell invasion in vitro. Ovarian cancer cells were treated with two independent *FSHR* (A and B) and *LHCGR* (A and B) siRNAs and negative control siRNA for 30 h. (**a**) OVCAR3 cell invasion, mean ± SEM from 3 independent experiments (*n* = 11–12). (**b**) COV362 invasion, mean ± SEM from 3 independent experiments (*n* = 10–12). (**c**) OVCAR3 invasion, mean ± SEM from 4 independent experiments (*n* = 10–16). (**d**) COV362 cell invasion, mean ± SEM from 2 independent experiments (*n* = 8). Data are shown as a percentage of the negative control siRNA. * *p* < 0.05, significantly different from control, Student’s t-test.

To determine whether FSHR and LHCGR promote ovarian cancer cell invasion, transient FSHR and LHCGR knockdown were performed in OVCAR3 and COV362 cells. Both *FSHR* A siRNA and *FSHR* siRNA B treated OVCAR3 cells showed significantly increased invasion compared to negative control siRNA treated cells (Figure 10a). Both *FSHR* A siRNA and *FSHR* siRNA B also increased invasion of COV362 cells although significance was only observed with *FSHR* A siRNA (Figure 10b). We showed by western blotting that both *FSHR* A siRNA and *FSHR* B siRNA treatment were able to significantly knockdown expression of the ~48 kDa band by ~40% (*p* = 0.019) and 52% (*p* = 0.007) in OVCAR3 cells and 40% (*p* = 0.016) and 35% (*p* = 0.041) in COV362 cells, respectively (Figure S4).

There was no significant difference in OVCAR3 invasion with *LHCGR* (A and B) siRNA treatment (Figure 10c) however, both *LHCGR* A siRNA and *LHCGR* B siRNA increased COV362 invasion (*p* < 0.0001 and *p* = 0.007) (Figure 10d). qRT-PCR confirmed that *LHCGR* A siRNA and *LHCGR* B siRNA significantly knocked down *LHCGR* mRNA by 75% (*p* = 0.0002) and 35% *(p* = 0.009) in OVCAR3 cells and by 45% (*p* = 0.005) and 58% (*p* = 0.002) in COV362 cells, respectively, compared to negative control siRNA (Figure S5a). *LHCGR* A siRNA and *LHCGR* B siRNA were able to knockdown *LHCGR* protein (~65 kDa band) by 43% and 64% in OVCAR3 cells and 66% and 43% in COV362 cells, respectively (Figure S5b,c).

## 3. Discussions

Our study is one of the few that has explored the effects of gonadotropin receptor knockdown in human serous ovarian cancer cell lines. We have shown that: (i) high *FSHR* or *LHCGR* mRNA expression is associated with early-stage and low-grade ovarian cancer. (ii) high *FSHR* or *LHCGR* mRNA expression is associated with increased PFS and OS in all ovarian cancer patients including high-grade disease, (iii) high FSHR expression in cancer cells and tumor associated blood vessels was associated with increased PFS and OS, (iv) both low FSHR and low LHCGR protein expression were associated with reduced PFS and OS in HGSOC patients, (v) FSHR knockdown increased invasion of OVCAR-3 cells and COV362 cells, and (vi) knockdown of LHCGR increased the invasion of COV362 cells. Together, these findings suggest that reduced FSHR and LHCGR expression are associated with dedifferentiation and poorer prognosis. The loss of FSHR and LHCGR expression can promote pro-metastatic cell behavior.

The findings in this study agree with previous studies demonstrating decreased *FSHR* mRNA and FSHR protein expression with increasing clinical stage and progression from borderline tumors to ovarian carcinomas [22] and higher *FSHR* expression in low grade tumors compared to grade III tumors [15,16]. Furthermore, using the Kaplan–Meier online plotter we showed that high *FSHR* expression was linked with increased PFS and OS in all ovarian cancer patients and patients with high grade disease. Additionally, high FSHR protein expression was associated with increased PFS and OS in patients with HGSOC. Contrastingly, a study in 2011 found high FSHR protein expression to be associated with reduced OS [17]. This study was conducted using a mixed ovarian cancer tissue cohort (*n* = 156) including various subtypes, clinical stage and tumor grade [17]. However, a larger study with HGSOC (*n* = 875) showed no relationship between FSHR expression and survival outcome [23]. A follow-up study by the same group investigating hormone receptor expression in a small cohort of low grade serous ovarian cancer (*n* = 26) did not observe a relationship with FSHR expression and patient survival [16]. These studies used a FSHR antibody from Abcam (ab150557) [16,23] and the same method for assessment used in the current study. However, the brief correspondence by Lenhard et al. did not include details of the FSHR primary antibody used or the method of assessment so it is difficult to compare the findings [17]. The contrasting findings in the FSHR studies are likely due to the different FSHR antibodies used and their ability to detect different FSHR isoforms in addition to the different methods of assessment. A major issue with FSHR antibody specificity has also been highlighted and only FSHR323 was found to be suitable for detecting FSHR by flow cytometry and immunohistochemistry [24]. A recent review article has highlighted the need for the critical analysis of FSHR expression in normal extragonadal and malignant tissues and the confirmation of immunohistochemistry data with additional methods [25]. Our immunohistochemistry study was conducted with the FSHR323 antibody [26,27] which first reported the expression of FSHR in tumor associated blood vessels [26]. Using this antibody, we confirmed the presence of FSHR in normal ovary, Fallopian tube and in tumor-associated BVs in HGSOC. Interestingly, we found that FSHR positivity in BVs was associated with both increased PFS and OS. We confirmed expression of FSHR in ovary and ovarian cancer cell lines using the FSHR323 antibody by Western blotting (Figure S3).

Our findings agree with previous studies that have reported significantly decreased *LHCGR* expression in malignant ovarian tumors compared to benign tumors and a steady decrease in *LHCGR* expression from low-grade to high-grade ovarian cancer [16,18]. Using Kaplan–Meier online plotter, we also observed a significant relationship between high *LHCGR* expression and increased PFS and OS in all ovarian cancers and patients with high grade disease. This agrees with a study in 1997 examining mRNA expression of *LHCGR* in early and advanced stage ovarian cancer (*n* = 43) demonstrating that negative *LHCGR* expression was associated with reduced OS (*p* = 0.047) [19]. In 2011, Lenhard et al. also found in a mixed cohort of ovarian cancer (*n* = 156) that patients with only LHCGR tumor positivity had improved OS compared to patients with only FSHR tumor positivity (*p* = 0.030) [17]. However, a larger study in HGSOC (*n* = 875) using LHCGR antibody (H-50, sc25828) showed no relationship between LHCGR with survival outcome [23]. We also found no relationship between PFS or OS and LHCGR expression alone in our HGSOC cohort using the same LHCGR antibody. However, patients that exhibited both low FSHR and LHCGR tumor IR scores had the poorest survival outcome. In contrast, patients that exhibited both high FSHR and high LHCGR tumor scores had the best survival outcome. Together, these findings suggest that gonadotrophin receptors may be markers of cellular differentiation and their loss in high grade tumors may be part of the de-differentiation process [15]. Wang et al., in 2003, suggested that during ovarian carcinoma progression in the presence of high FSH levels, cancer cells de-differentiate and lose dependence on FSH to promote cell growth [15]. It is feasible that LH and FSH may promote ovarian cancer proliferation in the presence of FSHR and LHCGR however, tumor cells lacking LHCGR or FSHR no longer depend on gonadotropins for their growth and exhibit more aggressive behaviour. This phenomenon also occurs in breast cancer where the loss of estrogen receptor levels is associated with more aggressive disease and tumors that are no longer dependent on estrogen for their growth [28].

Previous studies in HEK293N and granulosa cells support our hypothesis that low FSHR levels promote pro-tumorigenic cancer cell behaviour. Low plasma membrane concentration of FSHR in HEK293N cells selectively activated cyclic AMP (cAMP) independent ERK phosphorylation via β-arrestin dependent pathways [29]. This finding led to the notion that FSHR density at the plasma membrane might control signal transduction mechanisms to induce distinct biological effects [29]. Furthermore, ligand treatment of the human granulosa cell line hGL5 resulted in FSHR- and LHCGR-mediated ERK1/2 phosphorylation and cell proliferation due to receptor coupling to β-arrestins [30]. These findings indicate that expression of FSHR and LHCGR can be induced in hGL5 cells, but that the FSHR-dependent cAMP/PKA pathway is constitutively silenced [30]. This was proposed as a potential mechanism to protect cells from FSHR-cAMP-PKA-induced apoptosis [30]. More recent studies have addressed the physiological significance of FSHR mediated cAMP signalling in human ovarian follicles [31]. They found that the heteromerization of FSHR with the G-protein-coupled estrogen receptor (GPER) promoted oocyte survival by reprogramming the cAMP/death signals into proliferative stimuli [31]. Future studies are required to determine if low FSHR enables the heteromer formation with other membrane G protein-coupled receptors and lead to enhanced proliferation in ovarian cancer cells via β-arrestins dependent pathways.

Whilst *FSHR* mRNA was only detected in the positive control KGN cells and normal human ovary, FSHR protein expression was observed in KGN cells as well as in serous ovarian cancer cell lines. No *LHCGR* mRNA was detected in KGN cells whereas major protein bands were observed at ~75 kDa and ~100 kDa in KGN protein extracts. Discrepancy between expression of FSHR mRNA and protein has also been described for human umbilical vein endothelial cells [32] and endothelial cells in endometriosis samples [33]. The discord between *FSHR* and *LHCGR* with their respective protein levels is surprising but may be due to differences in mRNA turnover in cancer cells compared to gonadal tissues [25], mRNA instability [34], or varied protein half-lives in different cell types [35]. The discrepancy is unlikely due to the differential expression of the receptor isoforms as the qRT-PCR primers used for *FSHR* and *LHCGR* target exons 1–2 present in all FSHR and LHCGR isoforms.

In this study, FSHR protein bands were seen at ~48 kDa, ~55 kDa and ~65 kDa in the ovarian cancer cell lines and ovarian cancer tissue extracts using the FSHR H-190 antibody. The bands ~55 kDa and ~65 kDa are consistent with FSHR bands observed in normal human ovary using the same FSHR H-190 antibody [36]. We also observed bands between 50 and 65 kDa using the FSHR323 antibody, however, 87 kDa FSHR band was reported in human ovary and prostate cancer cells using this antibody [26,27]. Other literature show FSHR expression at ~70 kDa in mesenchymal stem cells [37], 75 kDa in OVCAR3, CAOV3 cells [38] and benign MCV152 cells [39], which are close to the ~65 kDa band seen in our study. Another study detected a 55 kDa band in follicular fluid, however, its significance was not reported [40]. The ~48 kDa band has not been observed in previous studies however, it is close to the FSHR 55 kDa isoform described in human peripheral blood monocytes [41]. We confirmed that the ~48 kDa band detected with FSHR H-190 was significantly reduced following treatment with FSHR siRNA. The reason for the discrepancy in FSHR molecular weight is not clear but is likely due to differences in antibody specificity [24] as well as differences in protein preparations and could also reflect the expression of different receptor isoforms. Splice variants of *FSHR* have been detected among animal species and in humans, three *FSHR* are translated into protein (see Figure S1) [42]. These FSHR isoforms can be formed via post-transcriptional, such as glycosylation and phosphorylation, which alters the receptor extracellular domain and impacts molecular weight [43]. It is also possible that FSHR may undergo a cancer induced proteolytic cleavage which has been described for other proteins [44]. The differences in the intensity of the 48 kDa and ~55 kDa in our protein preparations support this notion.

Posttranscriptional splice variants LHCGR have also been detected among animal species and in humans, five *LHCGR* splice variants are translated into protein (see Figure S1) [42]. LHCGR protein bands between 48 kDa and 92 kDa have been observed in human corpora lutea, Fallopian tube epithelium and placental mesenchymal cells [45]. Other studies have reported LHCGR expression in mouse ovary between 66 kDa and 70 kDa, which is close to the ~65 kDa and ~75 kDa bands seen in the serous ovarian cancer cell lines and normal human ovary in our study [46,47]. Full-length LHCGR-201 has a molecular weight of ~79 kDa and the band detected at ~75 kDa may be isoform LHCGR-204 [45]. The doublet bands seen in normal human ovary tissues are likely to be the LHCGR-201 and LHCGR-204 isoforms [45]. The 65 kDa band detected with LHCGR LS-C334599 was reduced following treatment with *LHCGR* siRNA to a greater extent than the 75 kDa band. A mass spectrometry approach would be useful to confirm and distinguish the different LHCGR and FSHR bands observed in the ovarian cancer cell lines and ovarian tissues.

We investigated the functional role of gonadotropin receptors in ovarian cancer by knocking down *FSHR* and *LHCGR* in OVCAR3 and COV362 cell lines and assessing their effects on cell invasion in vitro. In our study, OVCAR3 and COV362 cells treated with FSHR A siRNA had significantly increased invasion compared to the negative siRNA. The increased invasion following FSHR knockdown observed in serous ovarian cancer cells supports the findings observed in the FORKO mouse model which lacks FSHR [48,49]. The loss of FSHR in FORKO mice was associated with increased ovarian tumor development [48,49] and an increased migration of epithelial cells into the ovaries compared to wild-type mice [49]. FORKO mice were investigated at 12–15 months of age, which corresponds to the age that women are commonly diagnosed with ovarian cancer (~50–60 years of age). FSH and LH levels in the pituitary and plasma were significantly greater (up to 4-fold) in FORKO mice compared to wild-type mice suggesting that the absence of FSHR is associated with ovarian tumor development despite high gonadotropin levels [48]. However, other in vitro studies have found that FSHR knockdown was associated with decreased EMT markers in HO8910 and HEY cells [50], decreased cell proliferation and increased cell apoptosis in human ovarian mucinous cystadenocarcinoma cells [51]. On the other hand, FSHR overexpression increased cell proliferation and invasion in benign MCV152 cells [39] and non-tumorigenic OSE cell lines in the presence of FSH [52]. It is unclear why the FSHR overexpression studies disagree with our FSHR knockdown results and the FORKO mice studies. Limitations of our study include not adding gonadotropins to the culture system and assessing effects of proliferation following receptor knockdown.

To the best of our knowledge, LHCGR knockdown has not previously been conducted in human ovarian cancer cells. Studies in LHCGR knock out mice (LuRKO) have not reported increased ovarian tumor development but reported a bone phenotype [46]. LHCGR overexpression was associated with reduced proliferation, migration and invasion in SKOV3 ovarian cancer cells [53] supporting our findings that LHCGR knockdown promotes invasion.

## 4. Materials and Methods

### 4.1. Public Ovarian Cancer Transcriptomic Microarray Databases

*FSHR* and *LHCGR* expression were examined in publicly available ovarian cancer microarray databases. The CSIOVDB microarray expression database (http://csibio.nus.edu.sg/CSIOVDB/CSIOVDB.html) was used to examine the association of *FSHR* and *LHCGR* mRNA expression with the clinical features: clinical stage and tumor grade (*n* = 3431) [20]. The Kaplan–Meier online plotter tool for ovarian cancer (http://kmplot.com/analysis/): 2015 version database (*n* = 1305) was used to combine the mean expression of *FSHR* (Affymetrix ID: 211201_at) and *LHCGR* (Affymetrix ID: 207240_s_at) and generate PFS and OS curves [21].

### 4.2. Patient Tissue Cohort

The HGSOC patient cohort for the immunohistochemistry study consisted of tissue microarrays (TMA) (1 mm tissue cores) from patients diagnosed between 1988 and 2013 (*n* = 144). Table S1 summarises the clinicopathological characteristics of the HGSOC TMA patient cohort. Samples used for the Fluidigm qRT-PCR consisted of benign serous ovarian tumors (*n* = 17) and HGSOC patient tissues (*n* = 29, Table S2). Ethics approval was received from the Royal Adelaide Hospital Human Ethics Committee (RAH Protocol # 140201, Approval date 13 January 2014).

### 4.3. FSHR and LHCGR Immunohistochemistry

Immunohistochemistry was performed using a protocol adapted from a study in 2015 [54]. Archived formalin fixed paraffin embedded tissue sections (5 µm) were incubated on a heat plate at 60 °C. Sections underwent microwave antigen retrieval in 10 mM citric acid buffer, pH 6 at 100 °C for 10 min (Sixth sense, Whirlpool, Clayton, Australia), blocked with 5% goat serum for 30 min then incubated with the primary antibodies FSHR 323 (1/300 obtained from Prof Nicolae Ghinea [26] and LHCGR (H-50, 1/200, Santa Cruz) overnight at 4 °C (Table S3). Sections were then incubated sequentially with the secondary antibody: biotinylated goat anti-rabbit or goat anti-mouse (1/400, Dako, Sydney, Australia), followed by streptavidin-horse radish peroxidase (1/500, Dako, Australia) for 1 h at room temperature and peroxidase activity was detected using diaminobenzidine (DAB) and H_2_O_2_ (Sigma-Aldrich, St. Louis, MI, USA). Tissues incubated with no primary antibody and with rabbit or mouse immunoglobulins were included as negative controls. Normal ovary tissues from premenopausal women were used as positive controls.

### 4.4. Immunohistochemistry Assessment

The slides were scanned using the NanoZoomer (Hamamatsu Photonics, Hamamatsu City, Japan). The levels of FSHR and LHCGR intensity in the cancer cells and the percentage of positively stained cells were assessed using a manual scoring method as described previously [17]. Staining intensity was scored as strong (3+), moderate (2+), weak (1+), or negative (0). The percentage of positively stained tumor cells was graded between a score from 0 to 4; 0 = none, 1 (≤10% positive cells), 2 (11–50% positive cells), 3 (51–80% positive cells) and 4 (>80% positive cells) by were assessed manually by three independent researchers (JC, RA, CR). The immunoreactive (IR) score in the cancer cells was calculated by multiplying the intensity score with the percentage of positive cells. A low IR was defined as a score of ≤2+, and a high IR was a score of ≥3+ [17]. The staining intensity in blood vessels was scored as strong (3+), moderate (2+), weak (1+), or negative (0). A blood vessel intensity score of ≤1+ was defined as low and a score of ≥2+ as high.

### 4.5. Cell Lines

The human serous ovarian cancer cell lines: OVCAR3, CAOV3, and the granulosa tumor cell line (KGN) were purchased from American Type Culture Collection (ATCC, Manassas, VA, USA). COV318, OAW28 and COV362 were purchased from the European Collection of Authenticated Cell Cultures (ECACC). All cell lines were maintained in an environment of 37 °C and 5% CO_2_ as previously described [54].

### 4.6. Quantitative Real-Time PCR (qRT-PCR)

RNA was extracted from frozen tissue samples (stored at −80 °C) including benign ovarian tumor (*n* = 17) and HGSOC (*n* = 29) using TRIzol (Thermo Fisher). The presence of at least 50% cancer content was confirmed by hematoxylin and eosin staining and RNA quality was confirmed by agarose gel electrophoresis. cDNA was synthesised using iScript cDNA Synthesis kit (Bio-Rad) according to the manufacturer’s protocol. The Fluidigm gene expression qRT-PCR using a 96.96 Dynamic Array^TM^ integrated fluidic circuit (IFC) (Integrated Sciences) was performed at the Australian Cancer Research Foundation Cancer Genomics Facility, SA Pathology, South Australia. Firstly, 2 uL of each 20 X Taqman^®^ assay was pooled and the cDNA was preamplified with cycling conditions: 95 °C for 2 min followed by 14 cycles of 95 °C for 15 s and 60 °C for 4 min on the C1000 cycler (Bio-Rad). Using the IFC Controller MX (Fluidigm), the IFC was primed with control line fluid (Fluidigm) with the Prime (136 ×) script and the samples and assays were then loaded using the Load Mix (136 ×) script. Next, preamplified cDNA pre-mixed with 2 ×Quanta PerfeCTa qPCR Fast Mix, low ROX (Quanta BioSciences) and 20 ×GE Sample Loading Reagent (Fluidigm) along with 20 ×Taqman^®^ assays containing 2 ×Assay Loading Reagent (Fluidigm) were loaded in their respective inlets and cycling conditions proceeded on the BioMark^TM^ HD System (Fluidigm) as follows: 45 °C for 2 min, a Thermal Mix consisting of 70 °C for 40 min and 60 °C for 30 s, followed by a Hot Start of 98 °C for 1 min, then 35 cycles of 97 °C for 5 s and 60 °C for 20 s. BioMark^TM^ HD Data Collection software was used to collect gene expression data while the Fluidigm Real-Time PCR Analysis Software was used to visualise and export the data collected. Taqman^®^ assays used consisted of *FSHR* (Hs00174865_m1), *LHCGR* (Hs00896336_m1) and *TBP* (Hs00427620_m1). Cycle threshold (Ct) values were normalised to the housekeeper gene *TBP* using a comparative CT method [55]. Ct values for *TBP* were not significantly different amongst the tissue groups.

The cell lines were plated in triplicate at 3 × 10^5^ cells/well in 96 well plates and cultured for 24 h RNA isolation and reverse transcription were performed using the Taqman^®^ Gene expression Cells-to-CT™ kit (Applied Biosystems, Mulgrave, Victoria, Australia) as per manufacturer’s instructions as previously described [56]. The experiments were conducted using the Taqman^®^ primer sets for *FSHR* (Hs00174865_m1), *LHCGR* (Hs00896336_m1) (exon location shown in Figure S1) and βactin (4333762F) with the QuantStudio 12K Flex (Applied Biosystems). PCR cycling conditions were as follows: 50 °C for 2 min, 95 °C for 10 min followed by 40 cycles of 95 °C for 15 sec and 60 °C for 1 min. Samples with no RNA and no cDNA were included as negative controls. Ct values were normalised to the housekeeper gene β-actin using the 2^−∆∆CT^ method. Ct values for β-actin were not altered by siRNA treatment.

### 4.7. Western Blot

Protein extracts from ovarian cancer cell lines and ovarian tissue were prepared in RIPA buffer as described previously [56,57]. The protein extracts (5–40 µg) were electrophoresed on 4–20% TGX gels (Bio-Rad Laboratories, Hercules, CA, USA) and transferred onto Polyvinylidene Difluoride (PVDF) membranes (GE Healthcare, Little Chalfont, England, UK). The membranes were subsequently incubated with the primary antibodies (Table S2): FSHR (H-190, sc-13935, 1/400, Santa Cruz), FSHR (FSHR 323, 1/600), LHCGR (H-50, sc-25828, 1/400, Santa Cruz) and LHCGR (LS-C334599, 1/1000, LifeSpan Biosciences) for 2 h at room temperature and 1 h with anti-mouse IgG (1/4000, Sigma Aldrich) or anti-rabbit IgG (1/4000, Millipore, Burlington, MA, USA) peroxidase-conjugated secondary antibodies. Chemiluminescence (ECL Hyperfilm, GE Healthcare) was used to visualise protein expression. Membranes were scanned using ChemiDoc™ MP Imaging System (Bio-Rad Laboratories, Inc.) and analysed using Image Lab™ software (Version 6.0.1 build 34, Bio-Rad Laboratories, Inc.). β-actin anti-rabbit antibody (1/5000, Abcam cat Ab8227) was used as a loading control. Protein extracts were prepared as described previously [57] from normal ovaries from pre-menopausal women and advanced HGSOC tissues confirmed to express FSHR and LHCGR by immunohistochemistry, were included as positive controls.

### 4.8. FSHR and LHCGR siRNA Treatment

OVCAR3 and COV362 were plated in triplicate at 2 × 10^5^ cells/well in 6-well plates. The following day cells were transfected with *FSHR* and *LHCGR* siRNAs (Table S4, exon location shown in Figure S1) or negative control siRNA (10 nM, Ambion, 4390843, TX, USA) with Oligofectamine™ Reagent (1/100, Invitrogen, Life Technologies, Carlsbad, CA, USA), Opti-MEM media (Invitrogen, Life Technologies, Australia) and growth culture media (without antibiotics) for 30 h [56]. siRNA treated ovarian cancer cells were allowed to recover in growth culture media for 24 h before qRT-PCR and western blot to confirm knockdown efficiency (as described above) and invasion assays were performed.

### 4.9. Invasion Assays

Invasion assays were conducted as described previously [56]. OVCAR3 and COV362 cells transfected with siRNA (control, FSHR A, B and *LHCGR* A, B) were labelled with calcein-AM (1 µg/mL, Invitrogen) and 8 × 10^5^ cells/well were pipetted onto 12 µm filters (96-well plate, Chemo Tx, Neuro Probe, MD, USA) coated with Geltrex (0.6 µL/well, Life Technologies) for invasion assays. After 6 h, cells that migrated into the lower chamber were measured by fluorescence at 485–520 nm using the Triad series multimode detector (Dynex technologies, Chantilly, VA, USA). The knockdown efficiency of *FSHR*/FSHR and *LHCGR/LHCGR* expression was confirmed using qRT-PCR and/or western blot for each independent experiment.

### 4.10. Statistical Analyses

For the CSIOVDB microarray expression database statistical significance was evaluated using the Mann–Whitney U-test [20]. The Mann–Whitney U test or Kruskal–Wallis test was used to assess the Fluidigm qRT-PCR dataset. The Kaplan–Meier online plotter was used to calculate the hazard ratio (HR), 95% confidence intervals (CI) and log-rank *p* value and plot Kaplan–Meier survival curves using publicly available data [21]. Patients were grouped using the best cut-off selected by the Kaplan–Meier online plotter tool [21]. Time to relapse or death due to ovarian cancer in the TMA cohort was used as the endpoint in Kaplan–Meier (log rank test) and Cox regression analyses to determine whether FSHR or LHCGR IR score and FSHR or LHCGR intensity in blood vessels is associated with PFS and OS (IBM SPSS version 26). The Student’s *t*-test was used to determine statistical significance between siRNA control and FSHR or LHCGR siRNA groups (GraphPad Prism^®^ Version 7.0b). Statistical significance was accepted at *p* ˂ 0.05.

## 5. Conclusions

We showed that higher *FSHR* and *LHCGR* expression are associated with early stage, low grade ovarian cancer and that *FSHR* and *LHCGR* expression is reduced in HGSOC compared to benign ovarian tumors. HGSOC patients with both high FSHR and high LHCGR expression had best survival outcome, whilst patients with both low FSHR and low LHCGR had poorest survival outcome. Knockdown of FSHR or LHCGR expression increased invasion of serous ovarian cancer cells. The effects of gonadotropin receptor knockdown appear to be cell specific. Additional EOC cell lines, including other ovarian cancer subtypes and primary ovarian cancer cells, should be assessed in future studies. Together, our findings support the hypothesis that reduced FSHR and/or LHCGR is associated with a poorer prognosis and can promote pro-metastatic ovarian cancer cell behaviour.

## Figures and Tables

**Table 1:** Cox Regression analyses of the HGSOC patient cohort for FSHR and LHCGR immunostaining.

**a. Univariate Cox Regression Analyses for Progression-Free Survival and Overall Survival.**						
	**Progression-Free Survival**			**Overall Survival**		
**Variable**	**Relative Risk**	**95% CI**	***p*-Value**	**RELATIVE RISK**	**95% CI**	***p*-Value**
Age ^a^	1.48	0.97–2.25	0.066	1.34	0.85–2.10	0.204
Tumor stage ^b^	0.97	0.50–1.88	0.929	0.87	0.35–2.17	0.764
Tumor grade ^c^	0.94	0.56–1.56	0.805	0.96	0.56–1.65	0.888
Residual disease ^d^	1.72	0.90–3.30	0.101	2.39	1.12–5.11	0.024
FSHR ^e^	0.63	0.39–1.01	0.054	0.55	0.54–0.96	0.035
FSHR BV ^f^	0.39	0.23–0.68	0.001	0.40	0.23–0.73	0.001
LHCGR ^g^	0.85	0.66–1.09	0.206	0.83	0.62–1.11	0.199
LHCGR BV ^h^	0.76	0.47–1.21	0.248	0.93	0.57–1.51	0.756
FSHR and LHCGR groups ^i^		1.00		1.00		
3 vs. 2	0.62	0.36–1.06	0.081	0.52	0.28–0.94	0.032
3 vs. 1	0.37	0.17–0.83	0.016	0.30	0.11–0.77	0.013
**b. Multivariate Cox Regression Analyses for Progression-Free Survival and Overall Survival.** **All Variables Are Significant in Univariate Analysis**						
	**Progression-Free Survival (*n* = 79)**			**Overall Survival (*n* = 84)**		
**Variable**	**RELATIVE RISK**	**95% CI**	***p*-Value**	**Relative Risk**	**95% CI**	***p*-Value**
Residual disease ^d^	1.52	0.75–3.09	0.248	3.06	1.19–7.89	0.021
FSHR BV ^f^	0.37	0.19–0.70	0.002	0.48	0.25–0.89	0.02
FSHR and LHCGR groups ^i^	1.00			1.00		
3 vs. 2	0.76	0.41–1.40	0.381	0.40	0.14–1.09	0.073
3 vs. 1	0.58	0.23–1.48	0.255	0.50	0.25–0.98	0.044

a = Age a dichotomous variable, cut point <55 vs. ≥55; b = Tumor stage (FIGO stage III vs. FIGO stage IV); c = Tumors grade (moderate vs. poor); d = Residual disease status (negative vs. positive); e = FSHR (immunoreactive score) in cancer cells as a dichotomous variable, cut point ≤2 vs. ≥3; f = FSHR positivity in blood vessels (BV), as a dichotomous variable, cut point <1 vs. ≥1; g = LHCGR (immunoreactive score) in cancer cells as a dichotomous variable, cut point ≤2 vs. ≥3; h = LHCGR positivity in BV, as a dichotomous variable, cut point <1 vs. ≥1; i = FSHR and LHCGR groups, Group 1 = FSHR IR ≥3 and LHCGR IR ≥3; Group 2 = FSHR IR ≥3 and LHCGR ≤2 or FSHR IR ≤2 and LHCGR IR ≥3; Group 3 = FSHR IR ≤2 and LHCGR IR ≤2.

## Supplementary Materials

The following are available online at https://www.mdpi.com/1422-0067/22/1/71/s1. Table S1. Clinicopathological characteristics of the high serous ovarian cancer TMA cohort. Table S2. Clinicopathological characteristics of benign and high grade serous ovarian cancer cohort used in the Fluidigm qRT-PCR. Table S3. Summary of FSHR and LHCGR antibodies used for immunohistochemistry and western blotting. Table S4. *FSHR* and *LHCGR* siRNA used for knockdown studies. Table S5. Relationship between FSHR and LHCGR expression with clinicopathological parameters. Figure S1. Human *FSHR* and *LHCGR* splice variants. Figure S2. Follicle-stimulating hormone receptor (FSHR) and luteinising hormone receptor (LHCGR) expression in human ovary. Figure S3. Western blot using FSHR323 antibody. Figure S4. Effect of follicle-stimulating hormone receptor (*FSHR*) siRNA treatment on FSHR protein expression in OVCAR3 and COV362 cells. Figure S5. Effect of luteinising hormone receptor (*LHCGR*) siRNA treatment on LHCGR mRNA and protein expression in OVCAR3 and COV362 cells.
