# Supplementary material for: Reduced Gonadotrophin Receptor Expression Is Associated with a More Aggressive Ovarian Cancer Phenotype
Source: Int J Mol Sci. 2020 Dec 23;22(1):71. doi: 10.3390/ijms22010071 (PMC7793521; doi:10.3390/ijms22010071)
Supplement: Supplementary file 1 [file ijms-22-00071-s001.pdf]

Table S1. Clinicopathological characteristics of the high serous ovarian cancer TMA cohort.

**Serous ovarian carcinomas (n=144)**		
Age at diagnosis (years)	Median (range)	61 (24-87)
Histological grade	Moderate	22
	Poor	122
FIGO stage	Stage II	2
	Stage III	134
	Stage IV	8
Residual disease after surgery	No	21
	Yes	81
	Unknown	42
FSHR immunoreactive score	≤2	39
	≥3	73
	Lost/unstained cores	32
LHCGR immunoreactive score	≤2	82
	≥3	38
	Lost/unstained cores	24
Recurrence	No	30
	Yes	99
	Unknown	15
Cause of death	Ovarian cancer	87
	Other cause	14
	Alive	41
	Lost to follow-up	2

Table S2. Clinicopathological characteristics of benign and high grade serous ovarian cancer cohort used in the Fluidigm qRT-PCR

**Benign serous cystadenomas (n=17)**		
Age	Median (range)	60 (25-75)
**High grade serous ovarian carcinomas (n=29)**		
Age at Diagnosis	Median (range)	59 (38-84)
Histological Grade	Moderate<br>Poor	1<br>28
FIGO stage	Stage I<br>Stage II<br>Stage III	4<br>8<br>17

Table S3. Summary of FSHR and LHCGR antibodies used for immunohistochemistry and western blotting

Antibody	Source	Immunogen	Clonality	Dilution (IHC)	Dilution (WB)	Description
FSHR 323	In-house[26, 29]	Fusion protein amino acids 172-358	Monoclonal	1/300	1/600	IgG, mouse
FSHR (H-190)	Santa Cruz (sc-13935)	Peptide amino acids 1-190	Polyclonal	-	1/400	IgG, rabbit
LHCGR (H-50)	Santa Cruz (sc-25828)	Peptide amino acids 28-77	Polyclonal	1/200	1/400	IgG, rabbit
LHCGR (LS-C334599)	Sapphire Bioscience	Recombinant protein of human *LHCGR*	Polyclonal	-	1/1000	IgG, rabbit

Table S4. *FSHR* and *LHCGR* siRNA used for knockdown studies

**siRNA (Ambion), 10nM**	**ID#**	**Lot#**	**Target exons**
FSHR A	s5377	AS02B123	7/8
FSHR B	s5379	AS02B124	7/8
LHCGR A	s8163	AS02B125	2
LHCGR B	s8164	AS02BBRO	11

Table S5. Relationship between FSHR and LHCGR expression with clinicopathological parameters

	**Low FSHR**<br>**IR <3**	**High FSHR**<br>**IR ≥3**	**Low LHCGR**<br>**IR <3**	**High LHCGR**<br>**IR ≥3**
**Age**				
<55	13/39 (33%)	26/39 (67%)	30/42 (71%)	12/42 (29%)
≥55	24/71 (34%)	47/71 (66%)	53/77 (69%	24/77 (31.0%)
Chi-squared test[a]	*p*= 1.000		*p* = 0.837	
**Tumor stage**				
FIGO STAGE II	1/2 (50%)	1/2 (50%)	1/2 (50%)	1/2 (50%)
FIGO stage III	35/106 (33%)	71/106 (67%)	78/114 (68%)	36/114 (32%)
FIG0 stage IV	3/4 (75%)	1/4 (25%)	4/5 (80%)	1/5 (20%)
Chi-squared test	*p*= 0.202		*p*= 0.732	
**Tumor grade**				
Moderate	7/17 (41%)	10/17 (59%)	16/19 (84%)	3/19 (16%)
Poor	32/95 (34%)	63/95 (66%)	67/102 (66%)	35/102 (34%)
Chi-squared test[a]	*P* = 0.587		*P* = 0.177	
**Residual disease**				
No	4/16 (25%)	12/16 (75%)	9/19 (47%)	10/19 (53%)
Yes	20/70 (29%)	50/70 (71%)	58/74 (78%)	16/74 (22%)
Chi-squared test[a]	*P* = 0.837		***P* = 0.01**	

Fishers’s exact test

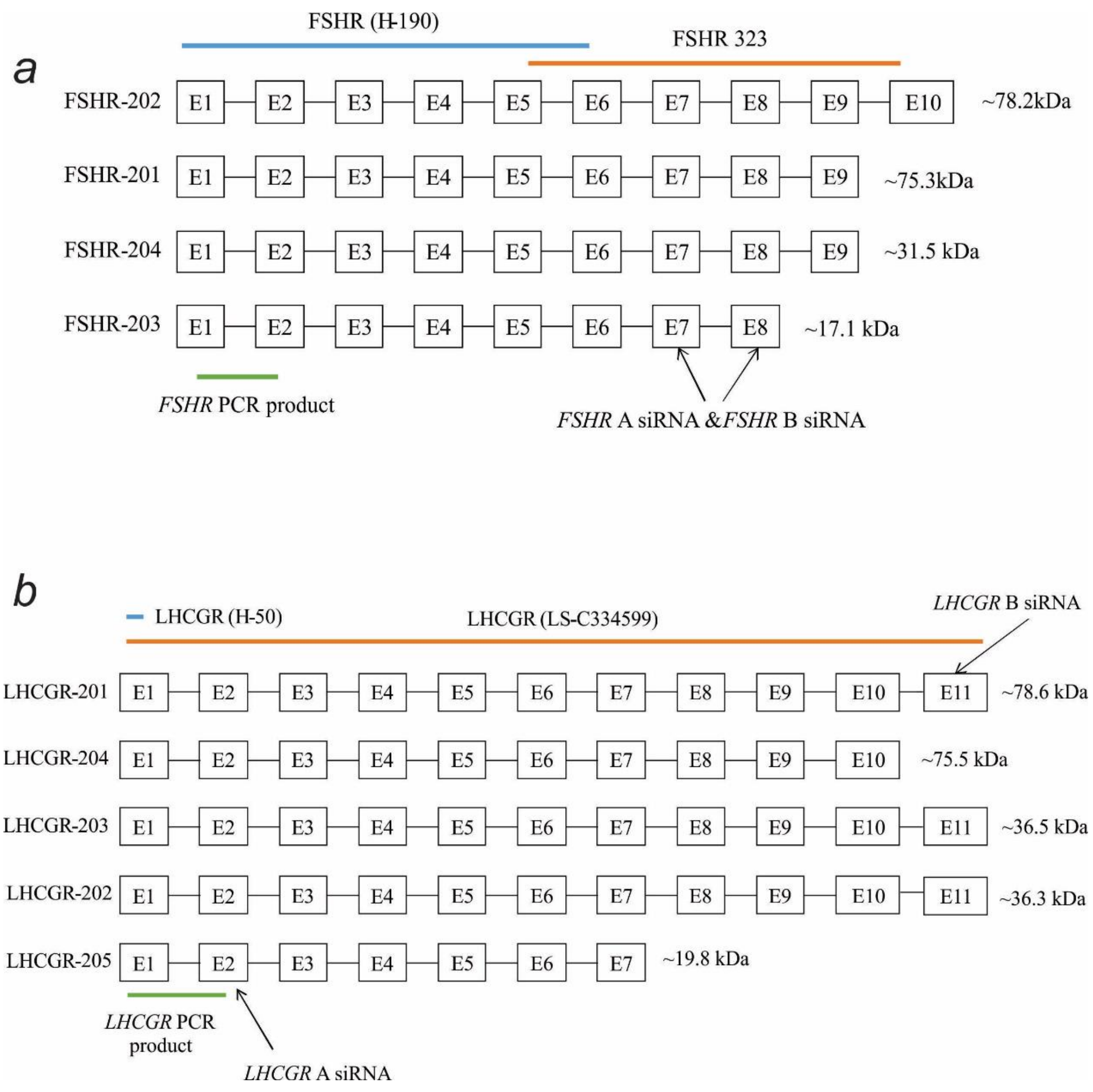

Figure S1. Human *FSHR* and *LHCGR* splice variants. E# refers to the exon number, not drawn to scale.. (*a*) FSHR siRNA target exons indicated by arrows. FSHR H-190 antibody target region shown by blue line. FSHR 323 antibody target region shown by red line. *FSHR* (Hs00174865_m1) qRT-PCR primer location shown by green line. FSHR sequences from FSHR Ensembl and NCBI reference sequence NM_000145.3. FSHR-203 is not protein coding (nonsense mediated decay) (*b*) LHCGR siRNA target exons indicated by arrows. LHCGR H-50 antibody target region shown by blue line. LHCGR LS-C334599 antibody target region shown by red line. *LHCGR* (Hs00896336_m1) qRT-PCR primer location shown by green line. LHCGR sequences from LHCGR Ensembl and NCBI reference sequence NM_000233.3. (https://asia.ensembl.org/index.html)

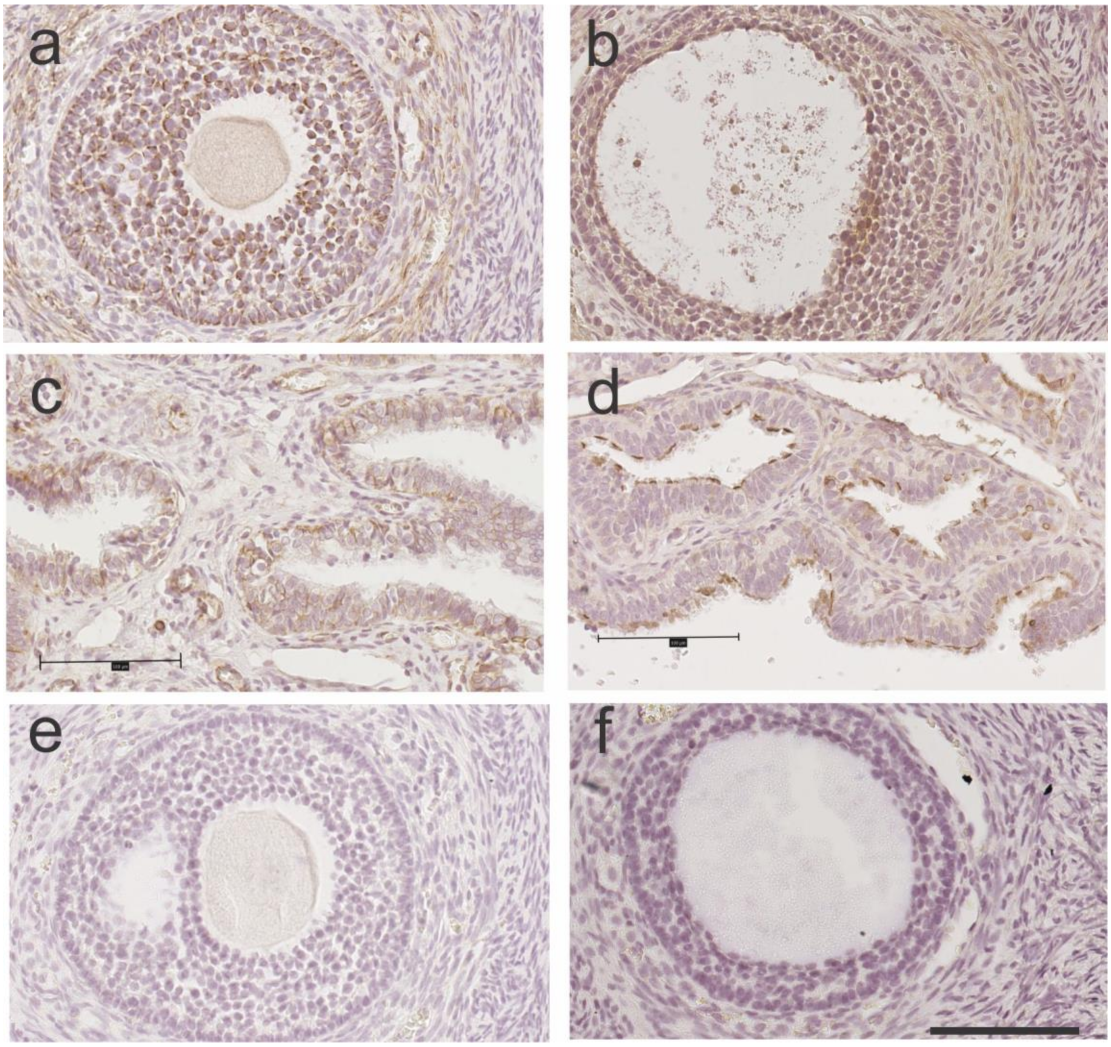

Figure S2. Follicle-stimulating hormone receptor (FSHR) and luteinising hormone receptor (LHCGR) expression in human ovary. (**a**) FSHR in normal ovary (FSHR 323 antibody, 1/300 obtained from Prof Ghinea [26]). (**b**) LHCGR in normal ovary and Fallopian tube (LHCGR H-50, 1/200, Santa Cruz). (**c**) FSHR in Fallopian tube (FSHR 323 antibody, 1/300). (**d**). LHCGR in Fallopian tube (LHCGR H-50, 1/200, Santa Cruz). (**e**) ovary with mouse IgG (3µg/ml) and (**f**) ovary with rabbit IgG (1µg/ml). Scale bar=100µm (all images the same magnification).

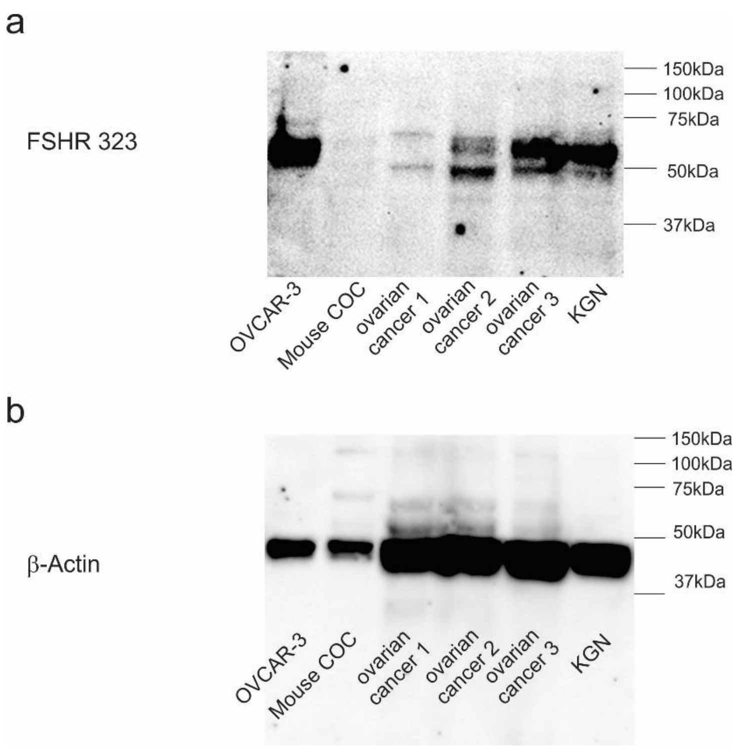

Figure S3. Western blot using FSHR323 antibody a) Protein extracts from cell lines (OVCAR3 and KGN, ~40µg) and ovarian cancer tissue extracts (~5µg) were electrophoresed and immunoblotted with FSHR323 (1/600) antibody. b) β-actin (1/2000, Abcam) was used as a loading control. FSHR bands were detected at ~50kDa and ~65kDa.

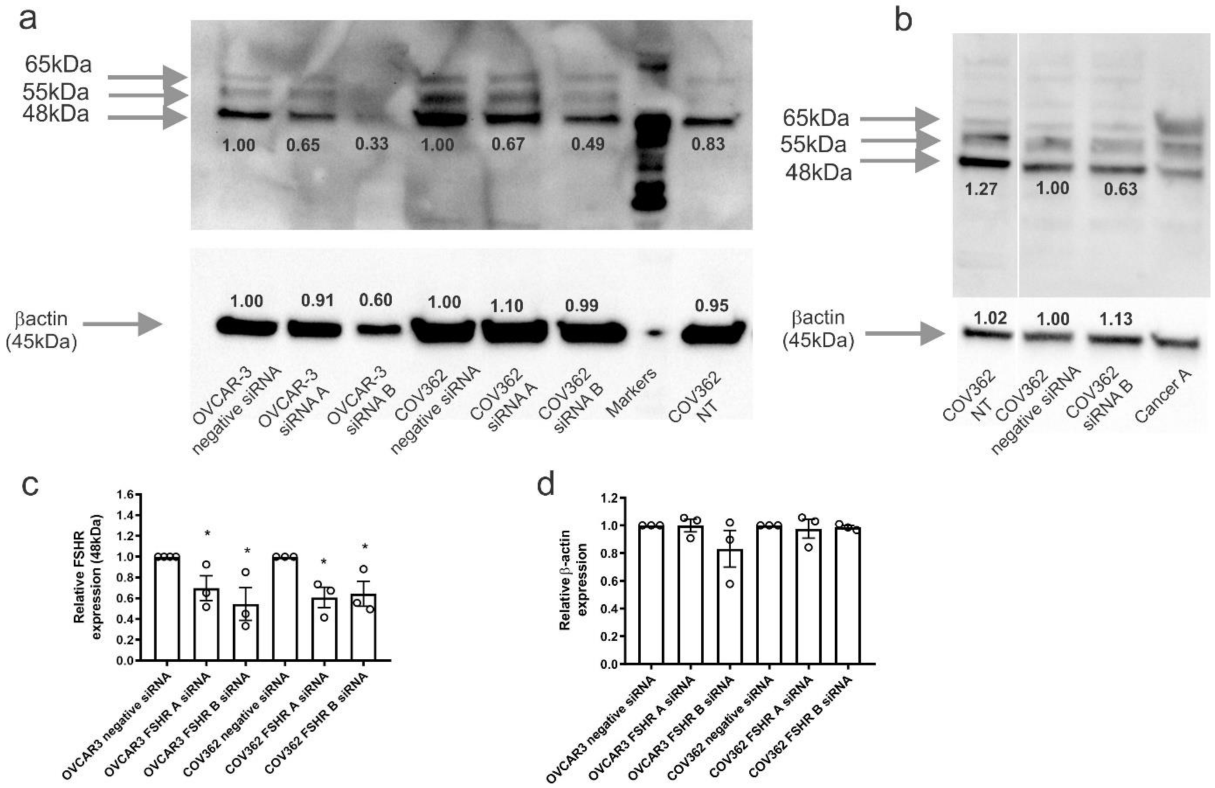

Figure S4. Effect of follicle-stimulating hormone receptor (*FSHR*) siRNA treatment on FSHR protein expression in OVCAR3 and COV362 cells. (*a*) Western blot following *FSHR* A and B siRNA treatment with FSHR (H-190) and β-actin antibodies in OVCAR3 and COV362 cells. ~40μg of protein for cell lines and ~5μg protein from ovarian cancer tissue extract were run on a 4-20% TGX gel and incubated with rabbit polyclonal antibodies FSHR H-190 (1/400, Santa Cruz) and β-actin (1/2,000, Abcam cat Ab8227). Numbers below protein bands are fold changes relative to the negative siRNA control treatment. **(*b*)** FSHR quantitation (48kDa) in *FSHR* A and B siRNA treated OVCAR3 and COV362 cells compared to the negative siRNA control treated cells. Data is from 3 independent experiments. Statistical significance was determined using the Student's t-test for the experiments on COV362 cells, *$p<0.05$. **(*c*)** FSHR and (d) β-actin expression in *FSHR* A and B siRNA treated OVCAR3 and COV362 cells compared to the negative siRNA control treated cells

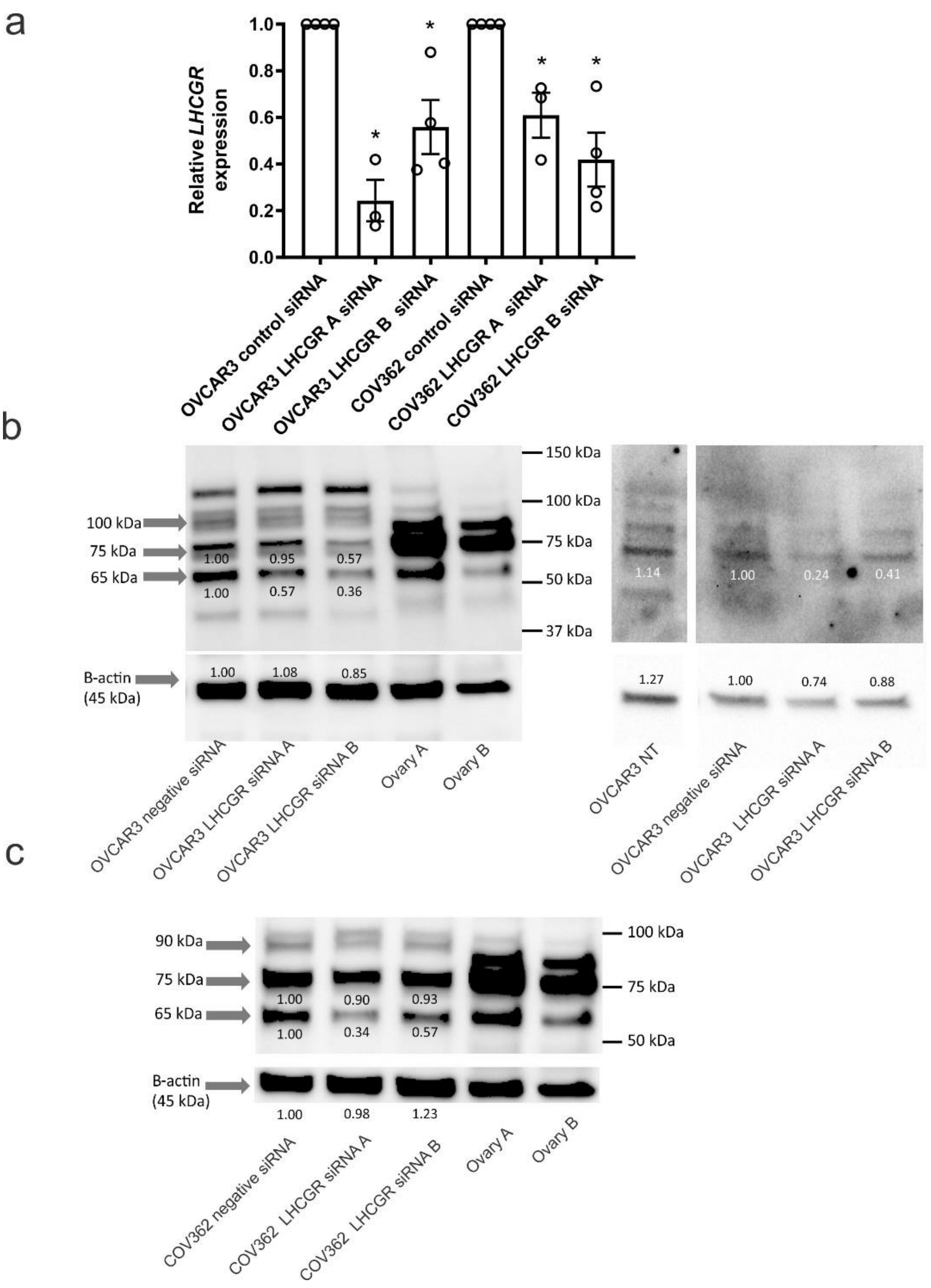

Figure S5. Effect of luteinising hormone receptor (*LHCGR*) siRNA treatment on LHCGR mRNA and protein expression in OVCAR3 and COV362 cells. (*a*) *LHCGR* expression was quantified using the $2^{-\Delta\Delta CT}$ method and normalised to housekeeping gene β-actin using negative siRNA control treatment as a calibrator. Data is from 3 to 4 independent experiments performed in triplicate (n=9 to n=12). Statistical significance from negative siRNA control treatment was determined using the Student's t-test, *p<0.05. Western blots for LHCGR expression following *LHCGR* A and B siRNA treatment in **(*b*)** OVCAR3 cells and **(*c*)** COV362 cells. Protein extracts (~40μg for OVCAR3 and COV362 & ~2μg for the normal ovary tissues) were run on a 4-20% TGX gel and incubated with rabbit polyclonal antibodies LHCGR LS-C334599 (1/1000, Sapphire Bioscience) and β-actin (1/2,000, Abcam cat Ab8227). Human ovary extracts were from pre-menopausal women. Numbers below protein bands are fold changes relative to the negative siRNA control treatment.
